# Overall treatment effects of aquatic physical therapy in knee osteoarthritis: a systematic review and meta-analysis

**DOI:** 10.1186/s13018-022-03069-6

**Published:** 2022-03-28

**Authors:** Ji Ma, Xiaoyu Chen, Juan Xin, Xin Niu, Zhifang Liu, Qian Zhao

**Affiliations:** 1 The Orthopaedic Spinal Ward, Shanxi Provincial People’s Hospital, 29th Shuangta Temple Street, Taiyuan, 030012 Shanxi People’s Republic of China; 2 School of Nursing, Shanxi University of Traditional Chinese Medicine, 121st Daxue Street, Yuci District, Jinzhong, 030619 Shanxi People’s Republic of China; 3 Department of Nursing, Shanxi Provincial People’s Hospital, 29th Shuangta Temple Street, Taiyuan, 030012 Shanxi People’s Republic of China

**Keywords:** Knee osteoarthritis, Aquatic physical therapy, Meta-analysis

## Abstract

**Objective:**

To determine the benefits of aquatic physical therapy as a rehabilitation strategy for knee osteoarthritis patients.

**Methods:**

Electronic databases systematically searched up to July 2021.

**Results:**

580 RCTs were selected. A total of thirteen studies comprising 883 participants were included in the study. For pain, meta-analyses showed that aquatic physical therapy is associated with a significant change in Western Ontario and McMaster University Osteoarthritis Index (WOMAC) pain (SMD = − 1.09, 95%CI − 1.97, − 0.21, *p* = 0.02) and visual analog scale (VAS) (SMD = − 0.55, 95%CI − 0.98, − 0.12, *p* = 0.01). In addition, for physical function, meta-analyses showed that aquatic physical therapy effectively improved WOMAC physical function (SMD = − 0.57, 95%CI − 1.14, − 0.01, *p* = 0.05). However, our findings showed no significant improvements in symptoms of joints, quality of life **(**QOL), flexibility, and body composition with knee osteoarthritis. For muscle strength, we found that aquatic physical therapy can only improve knee extension muscle strength (MD = 2.11, 95%CI 0.02, 4.20, *p* = 0.05). Additionally, for walking ability, we observed that aquatic physical therapy effectively reduced Timed-Up-and-Go Test (TUGT) in a large degree (MD = − 0.89, 95%CI − 1.25, − 0.53, *p* < 0.05).

**Conclusions:**

According to the findings reported in the studies analyzed in the review, aquatic physical therapy had a positive effect on the pain, physical function, knee extension muscle strength, and walking ability among people with knee osteoarthritis.

## Introduction

Osteoarthritis (OA) is the most prevalent form of arthritis and the main cause of disability in the older adults, and the knee is its most frequently affected weight-bearing joint [1]. This chronic and disabling condition not only reduces individual quality of life **(**QOL), but also exhausts a lot of health care resources and socioeconomic costs [1, 2]. Additionally, with the combined effects of aging, increasing obesity in the global population, and increasing numbers of joint injuries, the burden of osteoarthritis is becoming more common. According to global estimates, 250 million people are currently affected [3]. Therefore, there is an urgent need to explore methods of slowing down the progression of the disease.

The guidelines have strongly recommended that exercise is an effective non-pharmacological intervention for OA patients, which can relieve pain and enhance physical function [4]. Although both land and aquatic exercises can alleviate pain and improve the physical function of patients with OA [5], patients experience pain, stiffness, and muscle weakness during land exercises [6, 7], which limits their physical activity levels and leads them to a sedentary lifestyle [8, 9]. Correspondingly, lack of exercise will aggravate the progression of the disease.

In light of this, aquatic physical therapy would be an ideal form of physical activity for patients with OA. Because the buoyancy of water reduces the weight that joints, bones and muscles must bear [10], the warmth and pressure of water can also promote blood circulation and reduce joint pain and stiffness [11]. In addition, compared with other forms of treatments, aquatic physical therapy does not worsen joint condition [12] and leads to a higher level of treatment compliance [11]. And it is widely used as part of rehabilitation interventions for many diseases [13], such as rheumatic disease, fibromyalgia, stroke, and Parkinson disease [14–16].

There has been a meta-analysis of 11 trials for knee and hip OA patients and showed the positive effects of aquatic exercise on pain, stiffness, physical function, and QOL [17]. And another Cochrane review of 13 clinical trials also reached a similar conclusion [11]. However, a recent meta-analysis explored whether aquatic exercise is superior to land-based exercise in knee OA patients that showed comparable effects on the above outcomes [18]. Therefore, a consistent conclusion for the effect of aquatic physical therapy on knee OA alone could not be drawn [11]. Further, lack of sufficient evidence for the benefits of aquatic physical therapy, which limits recommendation on knee OA.

Although pain is the most prominent symptom of knee OA, it is often associated with other functional impairments, such as muscle weakness, reduced joint range of motion (ROM) and joint instability [12]. So the purposes of exercise in knee OA are not only to reduce pain and stiffness and restore impaired physical function and functional status, but also to improve ROM and maintain joint function and integrity. Additionally, Bliddal and Christensen reported that a 10% reduction in body weight could reduce OA symptoms by 28% [19], and it is necessary to investigate the effectiveness of aquatic physical therapy on body fat. Therefore, in addition to including pain, symptoms of joints, physical function, and QOL, we also included outcome measures of flexibility, muscle strength, walking ability, and body composition would provide a more comprehensive picture of the therapeutic value associated with aquatic physical therapy. For this purpose, we performed a systematic review and meta-analysis of randomized controlled trials (RCTs) to evaluate overall treatment effects of aquatic physical therapy in knee OA.

## Materials and methods

This is a meta-analysis of randomized trials involving the overall treatment effect of aquatic physical therapy in knee OA. The systematic review and meta-analysis were reported in accordance with the recommendations of the Preferred Reporting Items for Systematic Review and Meta-Analyses: The PRISMA Statement and Cochrane Handbook for Systematic Reviews of Interventions [20, 21]. The selected search strategy and methods of analysis were registered at the PROSPERO database (ref: CRD42021267364).

### Search strategy

We searched the following databases including Medline/PubMed, Web of Science, Embase, Cochrane Library and Chinese databases of the CNKI Scholar, VIP and WanFang. The relevant studies were searched from the inception of each database to July 2021. The search terms and strategy used were as follows: (hydrotherapy OR aquatic exercise OR water-based exercise) AND (osteoarthrosis OR arthritis degenerative OR arthritis) AND (randomized controlled trial OR RCT). Additionally, to search all relevant studies, the reference lists were also manually reviewed.

### Inclusion and exclusion criteria

The study inclusion criteria were as follows: (1) participants have a clinical diagnosis of knee OA; (2) participants aged ≥ 40 years; (3) participants have no medical conditions that prevent increased physical activities; (4) participants have not participated in an organized exercise program in the past 3 months; (5) during the intervention period, participants can actively participate in the treatment; (6) at least one group of intervention methods was aquatic physical therapy; (7) the study was reported at least one of the outcomes: pain, symptoms of joints, physical function, QOL, flexibility, muscle strength, walking ability, and body composition; (8) the type of study design was the RCT. Studies were excluded if (1) the type of article was conference abstracts, case reports, comments, letters to editor, review articles, or family-based studies; (2) the full text of the study was not available; (3) studies without available data; (4) the type of study design was not the RCT.

### Data extraction and quality assessment

Two independent researchers screened all abstracts identified in the initial search, excluded studies that violated the inclusion criteria, and removed all the duplicated references. If it was unclear whether the study met the selection criteria, advice could be sought from a third researcher and a consensus of opinion was made.

Information on first author and publication year, country, sample size, exercise type of experimental group and control group, intervention time, follow-up time and outcomes measures were extracted from the original reports. The quality of the trials included was assessed by the two independent researchers according to the Cochrane Collaboration Handbook recommendations and items such as: randomization, allocation concealment, blinding, incomplete outcome data and selective reporting [21].

It means low risk if the thesis clearly described, high risk if not described and unclear if described indeterminate in the text. Researchers achieved consensus by discussion, and if researchers didn't achieve, a third reviewer was consulted.

### Outcome measures

The main outcomes that were examined included: pain, symptoms of joints, physical function, QOL, flexibility, muscle strength, and walking ability and body composition. Across the studies, Western Ontario and McMaster University Osteoarthritis Index (WOMAC) pain, visual analog scale (VAS) score, and Knee Injury and Osteoarthritis Outcome Score (KOOS) pain were used to measure pain. Symptoms of joints were measured by the WOMAC stiffness and KOOS for symptoms. Physical function was measured by using the KOOS for activities of daily living (ADL), KOOS for sport/recreation, WOMAC physical function, and the medical outcomes study short form-36 (SF-36) physical function. QOL was measured by using the KOOS for QOL. Flexibility was measured by tests of joint range of motion (ROM) of knee extension and knee flexion. Knee extension and flexion and hip abduction muscle strength were used to measure muscle strength. Walking ability was quantified by the 6-min walk test (6MWT), walking speed, step test, or the Timed-Up-and-Go Test (TUGT). Body composition was evaluated by the body mass index (BMI) or the fat mass.

### Statistical analysis and risk of bias assessment

The data were analyzed by RevMan software (version 5.4.1). A meta-analysis intended to carry out RCTs, if the same outcomes had been assessed in at least two studies in a similar way, and at least one group received aquatic physical therapy. The mean difference (MD) and 95% confidence interval (CI) were calculated for continuous data to assess the change. For continuous outcomes with different scoring units, the standardized mean difference (SMD) with 95% confidence intervals (CI) was used to pool each outcome measure for estimating the effect size. The heterogeneity among studies was assessed by *I*^2^; if *I*^2^ < 50%, it could be considered that there was homogeneity among the trials, and the fixed-effects model was used; otherwise, a random-effects model was used (*I*^2^ ≥ 50%). A z test was adopted to test the combined effect and statistical significance was set at *p* < 0.05 [22]. In addition, subgroup analyses were used to compare the hip abduction muscle strength (left and right), and evaluation instruments (body composition: BMI and fat mass).

## Results

### Study selection and characteristics

**Fig. 1:**
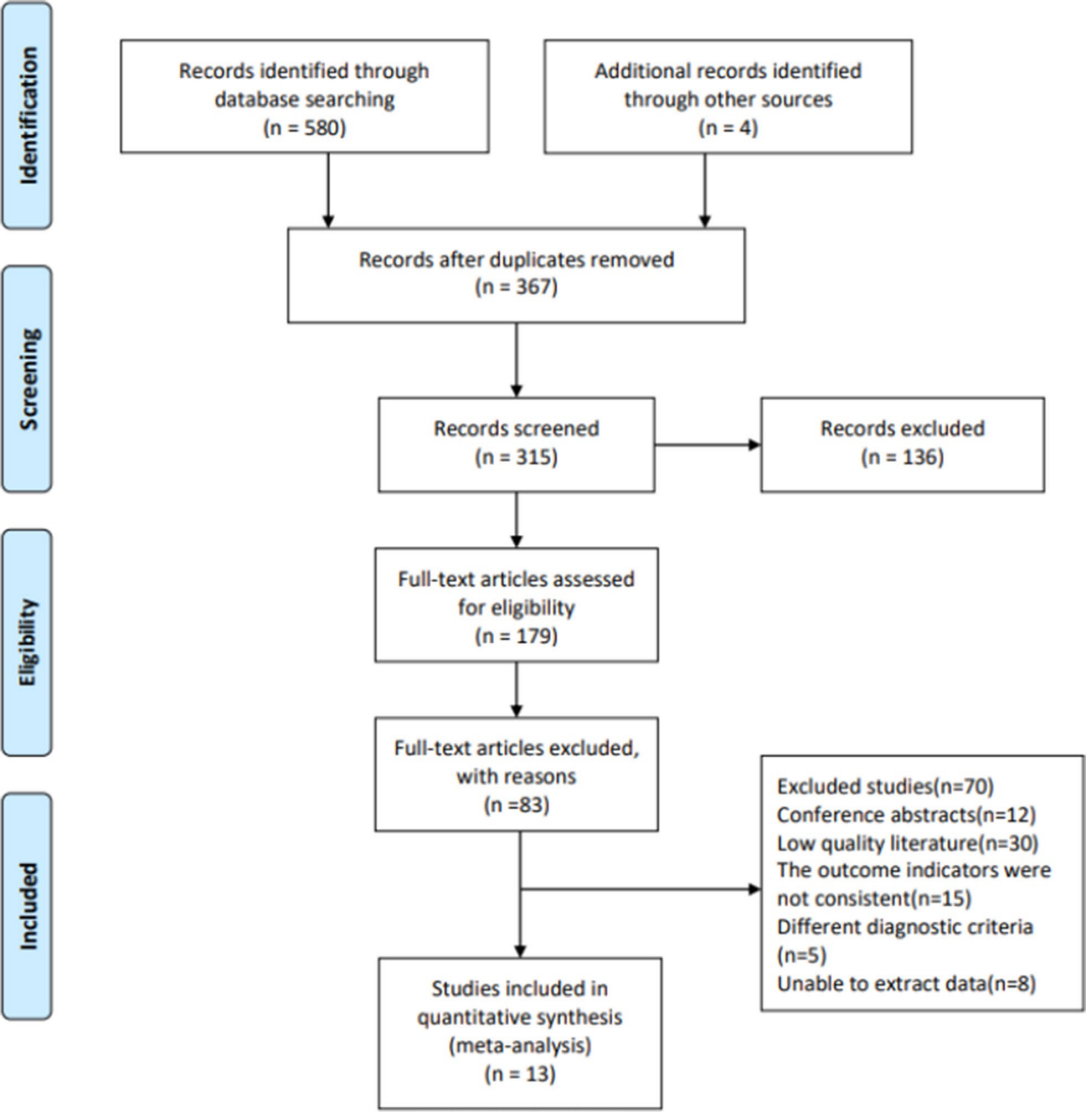
Flow diagram based on the Preferred Reporting Items for Systematic Reviews and Meta-Analyses (PRISMA) statement

A total of 580 studies were obtained by searching electrical databases, and thirteen trials [12, 23–34] were finally included (Fig. 1). There were 883 patients in total and involved for meta-analysis (357 aquatic physical therapy and 526 no aquatic physical therapy). A summary of characteristics of the included studies is shown in Table 1. All of the studies were published in English. Published in 2003–2019, the studies come from 10 different countries and regions. The duration of the interventional programs ranged from 6 to 18 weeks.

**Table 1:** Characteristics of studies included in the meta-analysis

First author (year)	Country of study	*n*_e1_/*n*_c1_ *n*_e2_/*n*_c2_	Experimental group (type of exercise)	Control group (type of exercise)	Intervention time	Outcomes measures
Dias [28]	Brazil	33/32	Aquatic exercise and an educational protocol	An educational protocol	Six weeks	WOMAC muscle strength Power and resistance
Silva [9]	Brazil	32/32	Aquatic physical therapy	Land-based exercise	18 weeks	Lequesne Index Scores WOMAC, VAS, 50FWT
Kars Fertelli [24]	Turkey	60/60	Aquatic physical therapy	Not receive any intervention	8 weeks	WOMAC, ASS Muscle strength
Hale [33]	New Zealand	23/16	Aquatic physical therapy	Computer skills training	12 weeks	Falls risk ratio Step test, TUGT, ABC Scale AIMS2-SF 26, WOMAC
Hinman [31]	Australia	36/35	Aquatic physical therapy	Usual care	6 weeks	VAS, WOMAC, AQOL, PASE Muscle strength step test, TUGT, 6MWT
Lim [32]	Korea	24/22 24/22	Aquatic physical therapy	Land-based exercise Home-based exercise	8 weeks	Body weight, BMI, lean body mass, body fat mass, body fat proportion, abdominal fat, BPI WOMAC SF-36 Peak torque, knee extensor and flexor
Lund [12]	Denmark	27/25 27/27	Aquatic physical therapy	Land-based exercise Not receive any intervention	8 weeks	VAS KOOS
Rantalainen [26]	Finland	42/42	Aquatic physical therapy	Usual care	16 weeks	T2 relaxation time, DGEMRIC index Cardiorespiratory fitness, force KOOS
Suomi [25]	WI	10/10 10/10	Aquatic physical therapy	Land-based exercise Not receive any intervention	8 weeks	Flexibility, hand–eye coordination Right arm curls, Left arm curls RSHab isometric, LSHab isometric, LHab isometric Functional capacity evaluation
Taglietti [34]	Brazil	31/29	Aquatic physical therapy	Educational program	8 weeks	VAS, WOMAC, SF-36 Depression, TUGT
Waller [27]	Finland	43/44	Aquatic physical therapy	Usual care	4 months	Walking speed, body mass, BMI, lean mass, fat mass KOOS
Wang [30]	USA	20/18	Aquatic physical therapy	Usual care	12 weeks	Flexibility, muscle strength 6MWT, MDHAQ, VAS
Wang [23]	Taiwan	26/26 26/26	Aquatic physical therapy	Land-based exercise Not receive any intervention	12 weeks	KOOS, ROM, 6MWT

WOMAC, Western Ontario and McMaster University Osteoarthritis Index; VAS, Visual Analog Scale; 50FWT, 50-foot (15.24-m) Walk Test; ASS, Arthritis Self-Efficacy Scale; TUGT, Timed-Up-and-Go Test; ABC, activity-specific balance confidence; AIMS2-SF, Arthritis Impact Measurement Scales 2-Short Form; AQoL, Assessment of Quality of Life Scale; PASE, Physical Activity Scale for the Elderly; 6MWT, 6-Min walk test; BMI, body mass index; BPI, brief pain inventory; SF-36, medical outcomes study short form-36; KOOS, Knee Injury and Osteoarthritis Outcome Score; T2, transverse relaxation time; DGEMRIC, delayed gadolinium-enhanced magnetic resonance imaging of cartilage; RSHab, right shoulder abduction; LSHab, left shoulder abduction; LHab, left hip abduction; MDHAQ, multidimensional Health Assessment Questionnaire; ROM, range of motion

### Critical appraisal

**Fig. 2:**
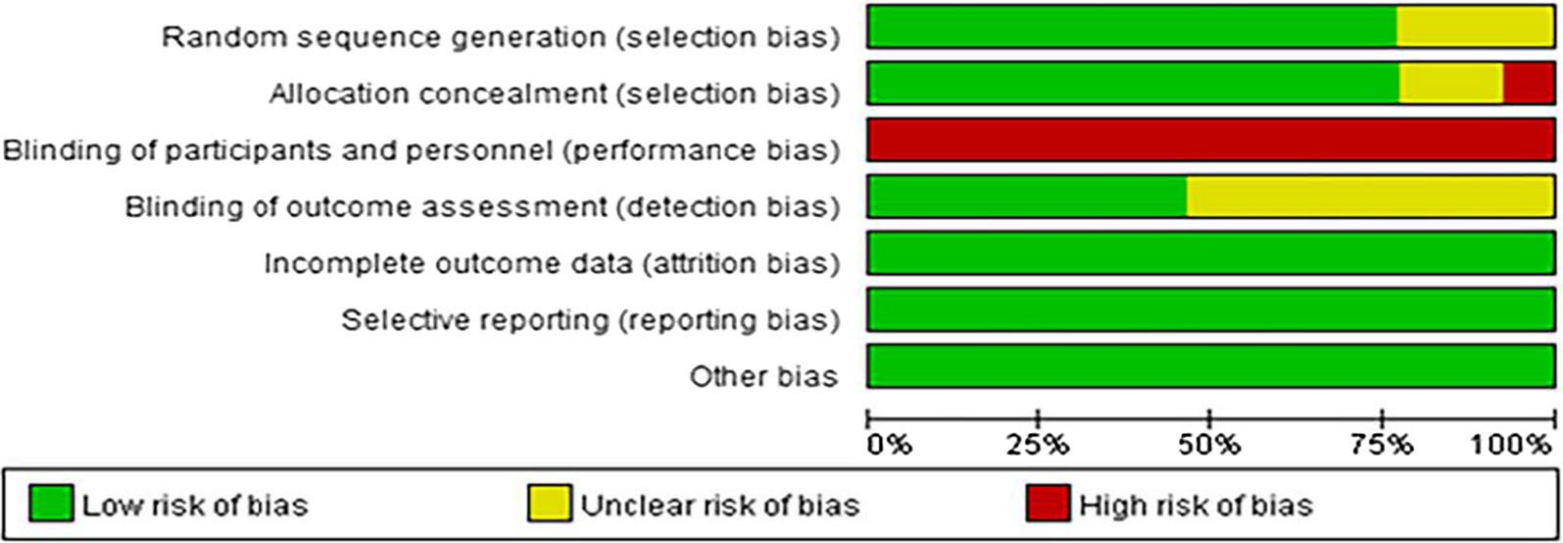
Risk of bias graph

**Fig. 3:**
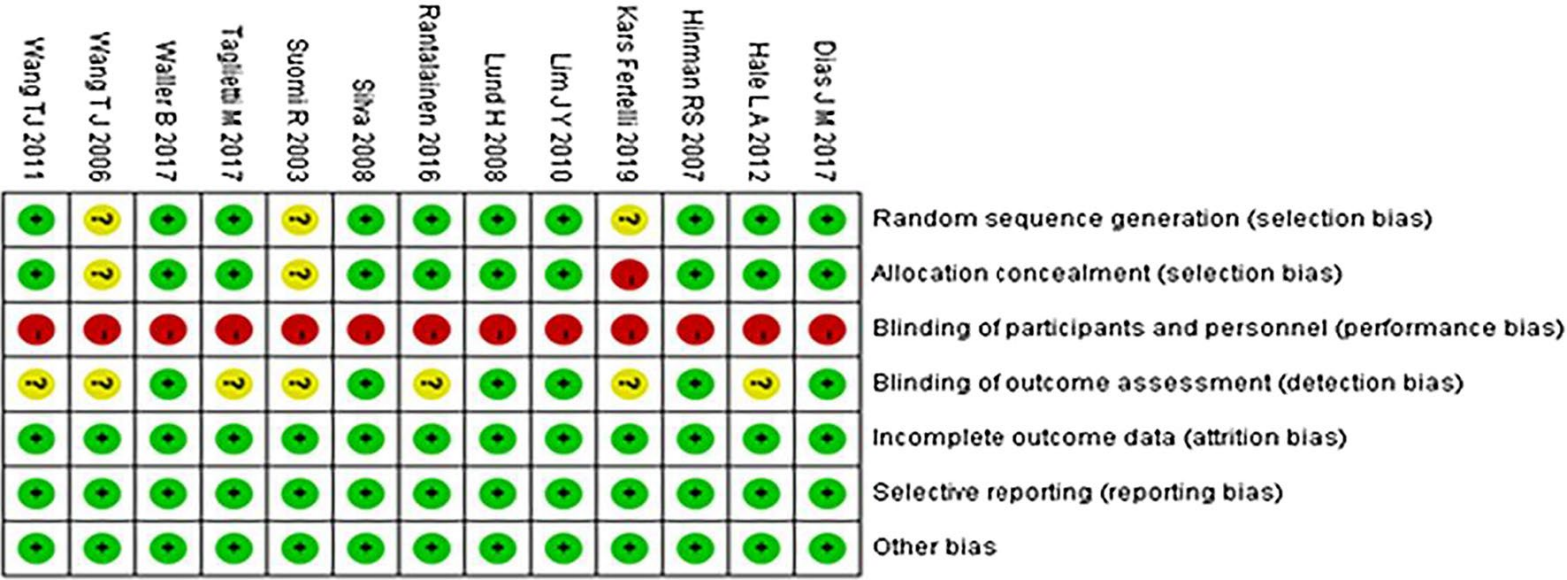
Risk of bias summary

The results of quality assessment of the included studies by Cochrane Collaboration Handbook are shown in Figs. 2 and 3. Ten had random sequence generation, ten had allocation concealment, no trials had blinding of participants and personnel, six had blinding of outcome assessment, no trials were assessed to have incomplete outcome data, and risk of selective reporting and other bias in all trials were low.

### Effect of intervention

#### Pain

**Fig. 4:**
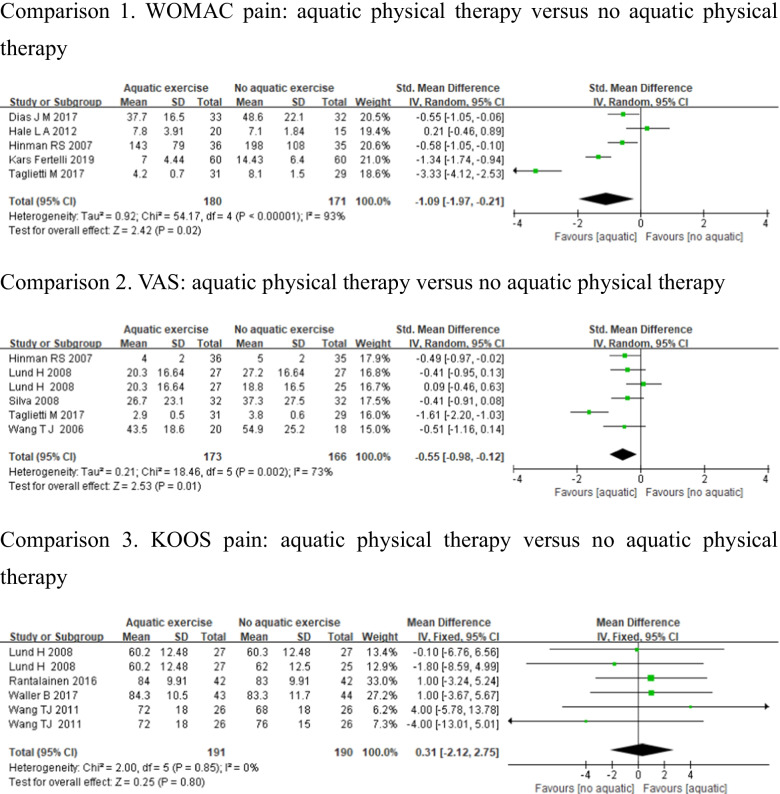
Forest plot of aquatic physical therapy versus no aquatic physical therapy interventions in pain

Eleven studies were included in the meta-analysis with outcome assessed pain. WOMAC pain [24, 28, 31, 33, 34], VAS score [12, 29–31, 34], and KOOS pain [12, 23, 26, 27] were used to measure pain. Studies which used WOMAC pain and VAS showed high heterogeneity (WOMAC pain: *p* < 0.1, *I*^2^ = 93%, VAS: *p* < 0.1, *I*^2^ = 73%), whereas KOOS pain showed low heterogeneity (*p* = 0.85, *I*^2^ = 0%). There were statistically significant differences in WOMAC pain (SMD = − 1.09, 95%CI − 1.97, − 0.21, *p* = 0.02), and VAS (SMD = − 0.55, 95%CI − 0.98, − 0.12, *p* = 0.01) in the aquatic physical therapy group compared to the no aquatic physical therapy group, but no significant difference in KOOS pain (MD = 0.31, 95%CI − 2.12, 2.75, *p* = 0.80) (Fig. 4).

#### Symptoms of joints

**Fig. 5:**
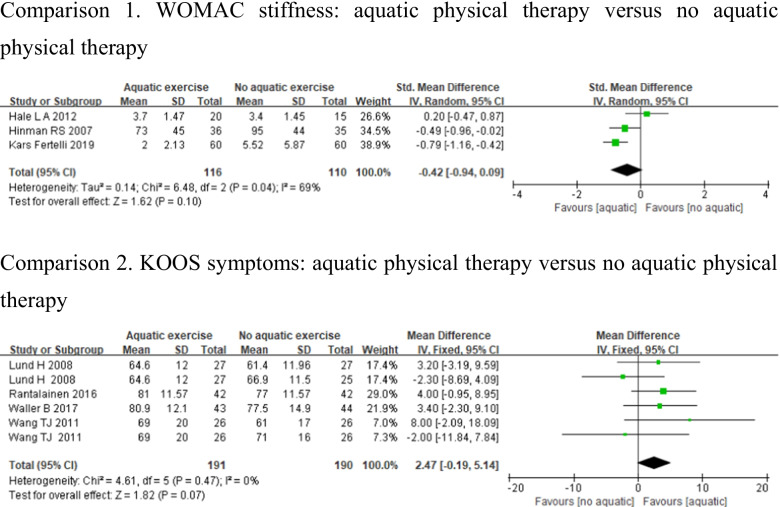
Forest plot of aquatic physical therapy versus no aquatic physical therapy interventions in symptoms of joints

Seven studies assessed symptoms of joints using WOMAC stiffness [24, 31, 33] and KOOS symptoms [12, 23, 26, 27]. Studies which used WOMAC stiffness showed substantial heterogeneity (*p* < 0.1, *I*^2^ = 69%), whereas KOOS symptoms showed low heterogeneity (*p* > 0.1, *I*^2^ = 0%). There were no significant differences in WOMAC stiffness (SMD = − 0.42, 95%CI − 0.94, 0.09, *p* = 0.1), and KOOS symptoms (MD = 2.47, 95%CI − 0.19, 5.14, *p* = 0.07) between aquatic physical therapy and no aquatic physical therapy (Fig. 5).

#### Physical function

**Fig. 6:**
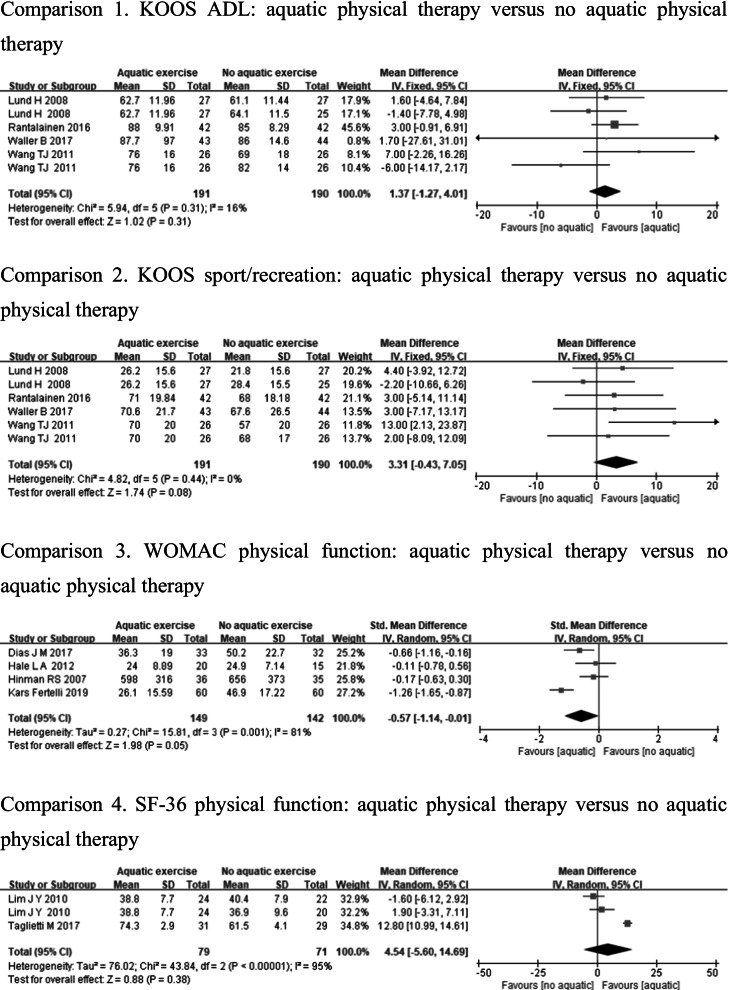
Forest plot of aquatic physical therapy versus no aquatic physical therapy interventions in physical function

Physical function was measured by using KOOS ADL [12, 23, 26, 27], KOOS sport/recreation [12, 23, 26, 27], WOMAC physical function [24, 28, 31, 33], and SF-36 physical function [32, 34]. Whereas KOOS ADL or KOOS sport/recreation showed low heterogeneity (KOOS ADL: *p* = 0.31, *I*^2^ = 16%, KOOS sport/recreation: *p* = 0.44, *I*^2^ = 0%), there were no significant differences in KOOS ADL (MD = 1.37, 95%CI − 1.27, 4.01, *p* = 0.31), and KOOS sport/recreation (MD = 3.31, 95%CI − 0.43, 7.05, *p* = 0.08) between aquatic physical therapy and no aquatic physical therapy. However, WOMAC physical function and SF-36 physical function demonstrated high heterogeneity (WOMAC physical function: *p* < 0.1, *I*^2^ = 81%, SF-36 physical function: *p* < 0.1, *I*^2^ = 95%), and there was statistically significant difference in WOMAC physical function (SMD = − 0.57, 95%CI − 1.14, − 0.01, *p* = 0.05) in the aquatic physical therapy group compared to the no aquatic physical therapy group, but no significant difference in SF-36 physical function (MD = 4.54, 95%CI − 5.60, 14.69, *p* = 0.38) (Fig. 6).

#### Quality of life

**Fig. 7:**
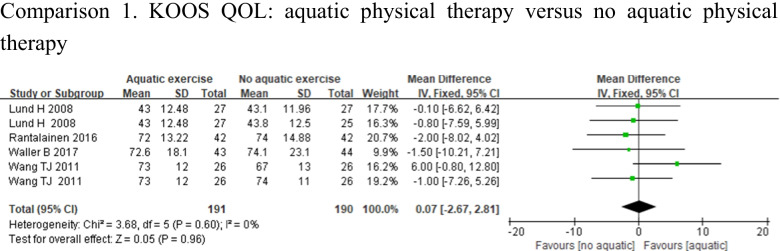
Forest plot of aquatic physical therapy versus no aquatic physical therapy interventions in quality of life

Four studies assessed QOL using KOOS QOL [12, 23, 26, 27]. Heterogeneity was not observed in the analyses for QOL (*p* = 0.6, *I*^2^ = 0%), and the meta-analysis (MD = 0.07, 95%CI − 2.67, 2.81, *p* = 0.96) demonstrated that there was no significant difference in the improvement of QOL between the 2 groups (Fig. 7).

#### Flexibility

**Fig. 8:**
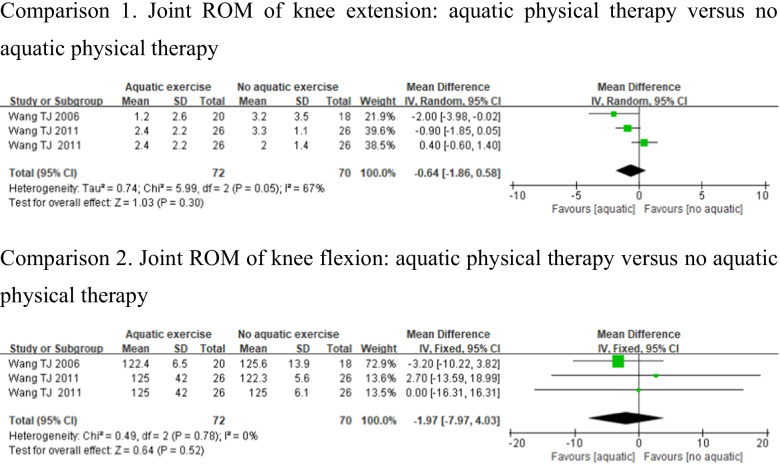
Forest plot of aquatic physical therapy versus no aquatic physical therapy interventions in flexibility

Flexibility was measured by tests of joint ROM of knee extension and knee flexion [23, 30]. Studies which used joint ROM of knee extension showed high heterogeneity (*p* = 0.05, *I*^2^ = 67%), whereas knee flexion showed low heterogeneity (*p* = 0.78, *I*^2^ = 0%). There were no significant differences in joint ROM of knee extension (MD = − 0.64, 95%CI − 1.86, 0.58, *p* = 0.30) and knee flexion (MD = − 1.97, 95%CI − 7.97, 4.03, *p* = 0.52) in the aquatic physical therapy group compared to the no aquatic physical therapy group (Fig. 8).

#### Muscle strength

**Fig. 9:**
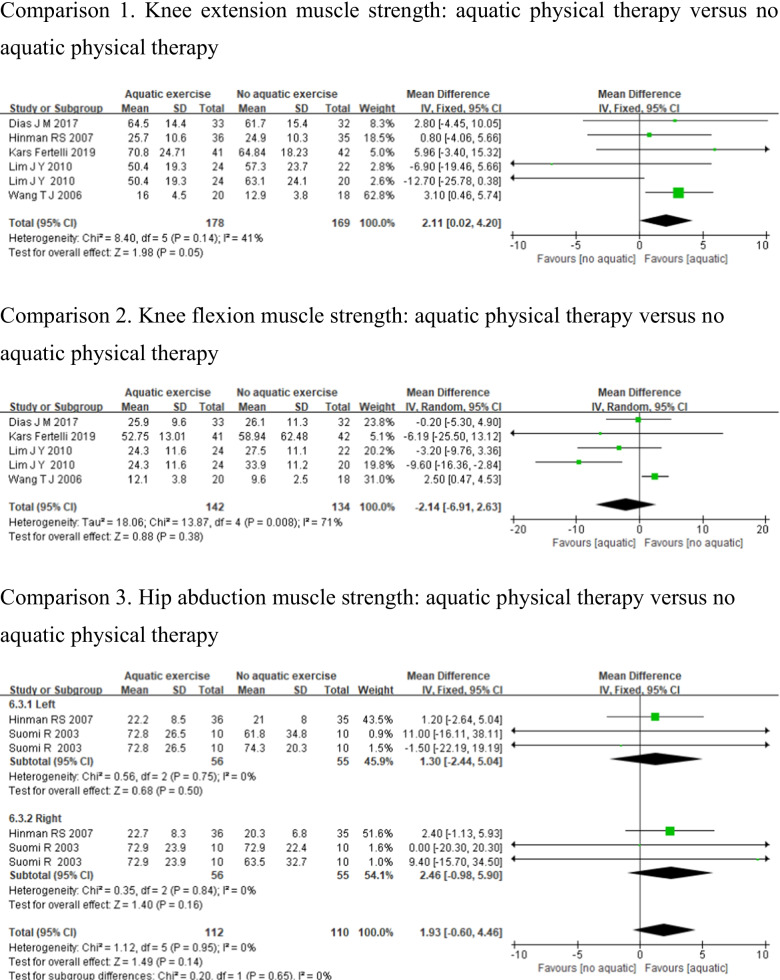
Forest plot of aquatic physical therapy versus no aquatic physical therapy interventions in muscle strength

Six studies were included in the meta-analysis with outcome measured muscle strength. Knee extension muscle strength [24, 28, 30–32], knee flexion muscle strength [24, 28, 30, 32], and hip abduction muscle strength [25, 31] were used to measure muscle strength. Due to the different muscle strength between the left and right sides in hip abduction studies, a subgroup analysis should be conducted for comparison. Heterogeneity was not apparent for knee extension (*p* = 0.14, *I*^2^ = 41%) and hip abduction muscle strength (left: *p* = 0.75, *I*^2^ = 0%, right: *p* = 0.84, *I*^2^ = 0%); however, knee flexion muscle strength demonstrated high heterogeneity (*p* < 0.01, *I*^2^ = 71%). And pooled analysis results demonstrate that aquatic physical therapy has no statistically significant differences than no aquatic physical therapy in improving knee flexion muscle strength (MD = − 2.14, 95%CI − 6.91, 2.63, *p* = 0.38), and hip abduction muscle strength (left: MD = 1.30, 95%CI − 2.44, 5.04, *p* = 0.50, right: MD = 2.46, 95%CI − 0.98, 5.90, *p* = 0.16). But there was a statistically significant difference in knee extension muscle strength between the 2 groups (MD = 2.11, 95%CI: 0.02, 4.20, *p* = 0.05) (Fig. 9).

#### Walking ability

**Fig. 10:**
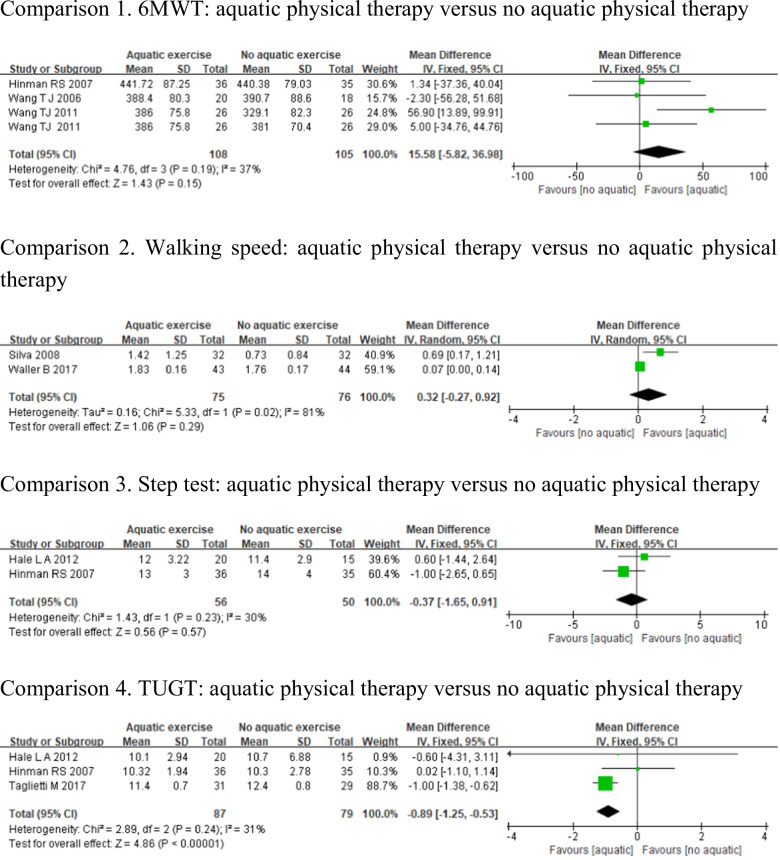
Forest plot of aquatic physical therapy versus no aquatic physical therapy interventions in walking ability

Walking ability was evaluated by 6-min walk test [23, 30, 31], walking speed [27, 29], step test [31, 33], and Timed-Up-and-Go Test [31, 33, 34]. Heterogeneity was not apparent for 6MWT (*p* = 0.19, *I*^2^ = 37%), step test (*p* = 0.23, *I*^2^ = 30%), and TUGT (*p* = 0.24, *I*^2^ = 31%); however, walking speed demonstrated high heterogeneity (*p* = 0.02, *I*^2^ = 81%). The aquatic physical therapy has no statistically significant difference in improving the scores of 6MWT (MD = 15.58, 95%CI − 5.82, 36.98, *p* = 0.15), walking speed (MD = 0.32, 95%CI − 0.27, 0.92, *p* = 0.29), and step test (MD = − 0.37, 95%CI − 1.65, 0.91, *p* = 0.57) compared to no aquatic physical therapy. But there was a statistically significant difference in TUGT between the 2 groups (MD = − 0.89, 95%CI − 1.25, − 0.53, *p* < 0.05) (Fig. 10).

#### Body composition

**Fig. 11:**
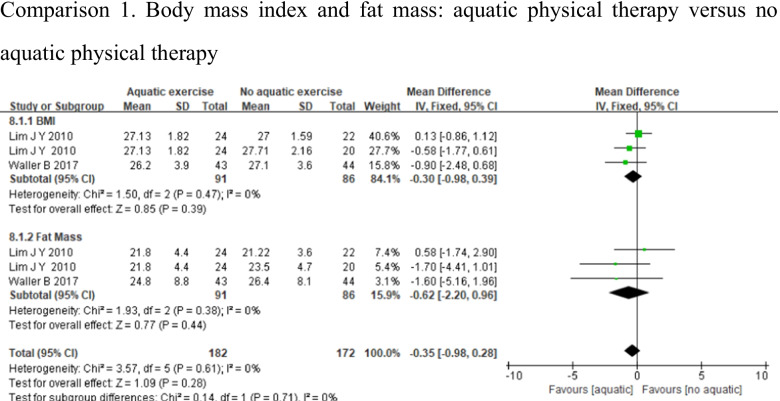
Forest plot of aquatic physical therapy versus no aquatic physical therapy interventions in body composition

Two studies assessed body composition using BMI [27, 32] and fat mass [27, 32]. Because the evaluation methods are different among these studies, a subgroup analysis should be conducted for comparison. Whereas BMI or fat mass showed low heterogeneity (BMI: *p* = 0.47, *I*^2^ = 0%, fat mass: *p* = 0.38, *I*^2^ = 0%), there were no significant differences in BMI (MD = − 0.30, 95%CI − 0.98, 0.39, *p* = 0.39), and fat mass (MD = − 0.62, 95%CI − 2.20, 0.96, *p* = 0.44) between the 2 groups (Fig. 11).

## Discussion

This systematic review and meta-analysis aimed to determine the overall treatment effect of aquatic physical therapy in patients with knee OA. Based on the included RCTs (*n* = 13), for pain, we found that aquatic physical therapy is associated with a significant change in WOMAC pain and VAS but not KOOS pain in people with knee OA. For symptoms of joints, our meta-analysis showed that aquatic physical therapy did not significantly relieve WOMAC stiffness and KOOS symptoms. Compared with no aquatic physical therapy, aquatic physical therapy cannot improve three test scores of physical function (KOOS ADL, KOOS sport/recreation and SF-36 physical function), but it has significant statistical differences in WOMAC physical function, and the effect size was moderate (WOMAC: SMD = − 0.57, 95%CI − 1.14, − 0.01, *p* = 0.05). Our findings also showed no significant improvements in QOL, flexibility, and body composition with knee OA. For muscle strength, we found that aquatic physical therapy can only improve knee extension muscle strength. In addition, for walking ability, we observed that aquatic physical therapy effectively reduced TUGT in a large degree. However, we also found that aquatic physical therapy cannot improve scores of the other three tests in walking ability (6MWT, walking speed, and step test). This may be due to the training was not intense or long enough, which is not enough to produce a significant statistical difference. Therefore, we concluded that aquatic physical therapy can improve pain, physical function, knee extension muscle strength, and walking ability to a certain extent.

Joint pain and stiffness are the most common symptoms in patients with knee OA and are the primary barriers for performing activities of daily living in this patient population [8]. Aquatic physical therapy is based on the buoyancy and temperature of water, which may encourage muscle relaxation, enhance greater movement to reduce joint and soft-tissue stiffness and, therefore, improve pain and physical function [31, 35]. Our study demonstrated that aquatic physical therapy can have a small and significant effect on pain and physical function, thus strengthening previous meta-analysis [11, 17, 36]. In addition, contrary to the previous findings [11, 17], our meta-analysis revealed that aquatic physical therapy cannot improve joints stiffness and QOL among people with knee OA. These differences in results can stem from the differences in the characteristics of the included studies. Therefore, our results may not accurately represent the true changes in joints stiffness and QOL within this population.

Meanwhile, the above changes were accompanied by the improvements in muscle strength and flexibility, as well as reductions in body composition. Muscle strength is clinically important as strong muscles act as shock absorbers and joint stabilizers, assisting to protect diseased joints [37]. The previous review [17] did not find any effect on muscle strength, whereas our study is the first to show that aquatic physical therapy can have a small but significant effect on knee extension muscle strength. The gradually and consistently increase in strength of knee extensor was a promising outcome of the program for preventing OA-associated disabilities in later life. The aquatic physical therapy, on the other hand, showed no effect on other major muscle groups, possibly due to too insufficient intervention intensity or duration to cause physiological changes in muscle structure [12].

A great improvement in walking ability of this study is a reduction in the TUGT, reflecting better control of the knee joint during walking and standing. Although the other three tests (6MWT, walking speed, and step test) used to evaluate walking ability have not been improved, this indicates that TUGT has greater specificity to patients with OA compared to the other three tests and consequently better responsiveness.

### Study limitations

However, some potential limitations of this study should be noted. First, more participants are needed to further study how aquatic physical therapy affects muscle strength of knee OA in a more systematic way. It may also be beneficial to follow the progress of participants to investigate the impact of aquatic physical therapy on knee OA patients over a longer period of time. Additionally, our review is unable to demonstrate the optimal intervention dose, type of exercise and training intensity for this population group.

## Conclusion

In conclusions, this meta-analysis confirmed that aquatic physical therapy is an effective treatment option for persons with severe symptoms of knee OA and should be considered as an important initial treatment option for rehabilitation programs. Researchers planning an aquatic physical therapy study should ensure that all aspects of the disease are considered, not just pain and physical function, and they need to refer to current recommendations when measuring results to promote the effectiveness of treatment. Future studies should aim to improve program content by maximizing the hydrostatic and hydrodynamic properties of water, so as to maximize the potential benefits of aquatic physical therapy for patients with knee OA.

## Data Availability

The datasets used and/or analyzed during the current study are available from the corresponding author on reasonable request.

## Abbreviations

ADL: Activities of daily living
BMI: Body mass index
KOOS: Knee Injury and Osteoarthritis Outcome Score
6MWT: 6-Min walk test
QOL: Quality of life
ROM: Range of motion
SF-36: Short form-36
TUGT: Timed-Up-and-Go Test
VAS: Visual analog scale
WOMAC: Western Ontario and McMaster University Osteoarthritis Index
